# Triple chronotherapy for the rapid treatment and maintenance of response in depressed outpatients: a feasibility and pilot randomised controlled trial

**DOI:** 10.1192/bjo.2021.199

**Published:** 2021-06-18

**Authors:** David Veale, Marc Serfaty, Clara Humpston, Andriani Papageorgiou, Sarah Markham, John Hodsoll, Allan H Young

**Affiliations:** 1 IoPNN King's College London, South London and Maudsley NHS Foundation Trust; 2 UCL Division of Psychiatry; 3 IoPPN King's College, Institute of Mental Health, University of Birmingham; 4 South London and Maudsley NHS Foundation Trust; 5 IoPPN King's College; 6 IoPPN King's College, South London and Maudsley NHS Foundation Trust

## Abstract

**Aims:**

Triple chronotherapy (defined as sleep deprivation for 36 hours, followed by 4 days of advancing the time of sleep, together with daily morning bright light therapy for 6 months) has demonstrated benefits for the rapid treatment of depressive symptoms in 4 small, controlled trials of in-patients. Our aims were to test the feasibility of recruitment and delivery of triple chronotherapy for out-patients with depression.

**Method:**

In a single blind trial, 82 participants were randomised to either triple chronotherapy or a control intervention. The primary outcome was Hamilton Depression Rating Scale 6 item (HAM-D6) at 1 week. Timings of observer ratings were baseline; 1 week; 2 weeks; 4 weeks; 8 weeks and 26 weeks after randomisation. Triple chronotherapy consisted of (a) Total sleep deprivation for 36 hours. On Day 1 patients were supported in a small group to stay awake at night with an occupational therapist, (b) Phase Advance of Sleep over 4 days. Phase Advance began after the first night of sleep deprivation, when they left the hospital at about 8am and were asked to go to bed earlier at about 5pm and rise at about 1am. Their sleep and wake up times were then shifted 2 hours later on each of the following three days until they attained their usual bedtime again at about 11pm.As a control for the triple chronotherapy, participants were given psychoeducation and written information on sleep hygiene. They were also given SomniLight amber light daily for 1 week in the morning.

**Result:**

Participants in the triple chronotherapy group were able to stay awake for the planned thirty-six hours and 89.9% adhered to the plan of phase advance of their sleep over the following 4 days. We achieved our recruitment target with 60 participants having completed the trial within 13 months. There were no reported adverse side effects. We explored outcomes and found a significant difference between the groups for the HAM-D6 at week 1, 8 and 26. Response (> 50% reduction in symptoms) was achieved by 52% in the triple chronotherapy group compared to 18% in the control group at week 1. This gradually increased to 70% achieving response in the triple chronotherapy group at week 26 compared to 22% in the control group.

**Conclusion:**

Triple chronotherapy produced a significant and rapid benefit after 1 week in out-patients with depression that was sustained at 26 weeks. Further cost-effective trials with a larger clinical sample size are required.

